# Chlojaponilactone B Attenuates THP-1 Macrophage Pyroptosis by Inhibiting the TLR/MyD88/NF-κB Pathway

**DOI:** 10.3390/ph17030402

**Published:** 2024-03-21

**Authors:** Qiyin Wen, Bingjinfeng Zhan, Lu Jin, Zijing Peng, Ju Liu, Longping Zhu, Depo Yang, Xinjun Xu, Lixia Zhang, Ge Li, Zhimin Zhao

**Affiliations:** 1 School of Pharmaceutical Sciences, Sun Yat-sen University, Guangzhou 510006, China; 2 Yunnan Key Laboratory of Southern Medicine Utilization, Yunnan Branch Institute of Medicinal Plant Development, Chinese Academy of Medical Sciences, Jinghong 666100, China

**Keywords:** Chojaponilactone B, lindenane sesquiterpenoid lactone, NLRP3 inflammasome, pyroptosis, anti-inflammation, *Chloranthus*

## Abstract

Pyroptosis, an innate immune response, plays a crucial role in the pathological process of inflammatory diseases. Although pyroptosis blockade is considered a potential therapeutic strategy, no ideal candidate drug has been identified. The natural product Chojaponilactone B (CJB) has demonstrated anti-inflammatory effects, but its role in macrophage pyroptosis has not been studied. This study aimed to investigate the effect and mechanism of CJB in inhibiting macrophage pyroptosis. Using an LPS/ATP-induced THP-1 macrophage pyroptosis model, we found that CJB significantly inhibited pyroptosis and reduced the levels of NLRP3, caspase 1, N-GSDMD, and inflammatory cytokines IL-1β and IL-18. RNA sequencing analysis revealed that CJB interfered with LPS/ATP-induced THP-1 macrophage gene expression, suggesting involvement in anti-inflammatory and anti-pyroptotic signaling pathways. Additionally, CJB suppressed LPS/ATP-induced elevations in TLRs, MyD88, pro-IL-1β, and NF-κB and blocked NF-κB p65 nuclear translocation. In summary, CJB inhibits NLRP3 activation and macrophage pyroptosis through the TLR/MyD88/NF-κB pathway, providing important evidence for its development as a potential drug for treating pyroptosis-related inflammatory diseases.

## 1. Introduction

Pyroptosis is a programmed cell death different from apoptosis, and can be triggered by microbial infection and various influencing factors [1]. This process is characterized by cell swelling, the formation of holes in the cell membrane, plasma membrane rupture, and the leakage of cellular contents, ultimately leading to cell lysis. Accompanied by the release of inflammatory mediators, pyroptosis amplifies the inflammatory response [2,3]. Pyroptosis was initially defined as caspase 1-dependent cell death, also known as canonical pyroptotic death. Under the stimulation of external conditions, cytosolic pattern recognition receptors, the precursor caspase 1, and the apoptosis-associated speck-like protein (ASC) assemble to form a canonical inflammasome [4]. This assembly activates caspase 1, which then cleaves the precursors of interleukin-1β (IL-1β) and interleukin-18 (IL-18), as well as gasdermin D (GSDMD) [5]. The cleaved form of GSDMD, specifically N-GSDMD, creates pores in the cell membrane, facilitating the release of proinflammatory cytokines outside the cell [6]. However, recent research has identified an alternative, non-canonical pathway of pyroptosis. In this pathway, GSDMD can also be cleaved by caspases 4/5/11, triggering pyroptosis [7,8]. This alternate mechanism enhances our understanding of the regulation and execution of pyroptosis, revealing its complexity and diversity. This alternate pathway adds to the complexity of pyroptosis, demonstrating its diverse regulatory mechanisms and execution processes.

Pyroptosis plays a pivotal role in various inflammatory diseases [9,10,11]. Of particular interest, macrophages, as central players in inflammation and immune processes, exhibit a significant impact on the progression of these diseases through their pyroptotic state. For instance, in atherosclerotic disease [12,13], a type of inflammatory disease affecting the arterial wall, nicotine can induce macrophage pyroptosis, thereby promoting disease progression [14]. Furthermore, GSDME-mediated macrophage pyroptosis has also been found to be closely associated with the development of atherosclerosis [15]. In the treatment of arthritis, the monomer derivative of paeoniflorin has been shown to alleviate disease symptoms by inhibiting macrophage pyroptosis [16]. Recent studies have also suggested that inhibiting macrophage pyroptosis may help to alleviate pulmonary inflammation and fibrosis [17]. As aforementioned, macrophage pyroptosis plays a pivotal role in infection, immune response, and inflammatory diseases, particularly in its close association with conditions such as cancer, arthritis, and cardiovascular diseases. Consequently, targeting the regulation of macrophage pyroptosis holds the potential to provide novel and effective therapeutic strategies for the treatment of these inflammatory disorders.

Chlojaponilactone B (CJB) is a lindenane-type sesquiterpenoid lactone isolated from the medicinal plant *Chloranthus japonicus* [18]. Previous studies have demonstrated the remarkable anti-inflammatory effects of CJB, with a particular emphasis on its outstanding capacity to suppress the production of nitric oxide (NO) induced by LPS in RAW264.7 cells. CJB has also been shown to significantly reduce the secretion of inflammatory mediators such as iNOS, COX-2, TNF-α, and IL-6 [19]. Furthermore, in animal experiments, CJB has successfully alleviated ear swelling in mice induced by 12-O-Tetradecanoylphorbol-13-acetate [20]. Despite extensive research into the anti-inflammatory properties of CJB, its role in the process of pyroptosis remains incompletely understood. Pyroptosis, a unique form of cell death, is closely associated with inflammatory cascade reactions. Given the anti-inflammatory characteristics of CJB, the present study aims to investigate its anti-pyroptotic activity using an LPS/ATP-induced THP-1 macrophage pyroptosis model. This study will focus on how CJB affects the activation of NOD-like receptor protein 3 (NLRP3) and will seek to reveal the underlying molecular mechanisms by which CJB regulates macrophage pyroptosis. This research has the potential to provide novel insights into the pharmacological effects of CJB and may offer valuable clues for the development of novel anti-pyroptotic drugs.

## 2. Results

### 2.1. CJB Suppressed the Pyroptosis of THP-1 Macrophages

**Figure 1:**
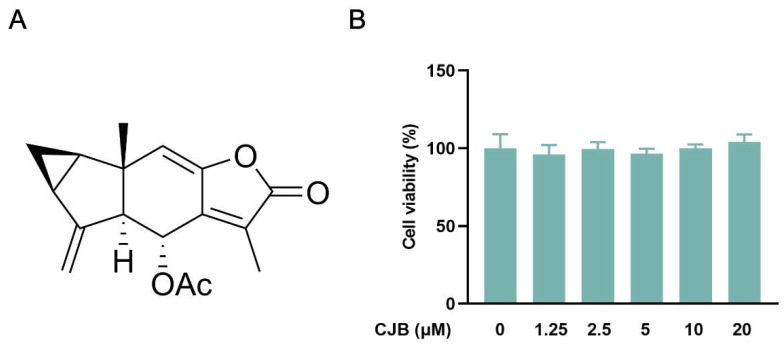
(**A**) Structure of CJB. (**B**) The cell viability of THP-1 macrophages after CJB (0–20 μM) treatment for 24 h.

**Figure 2:**
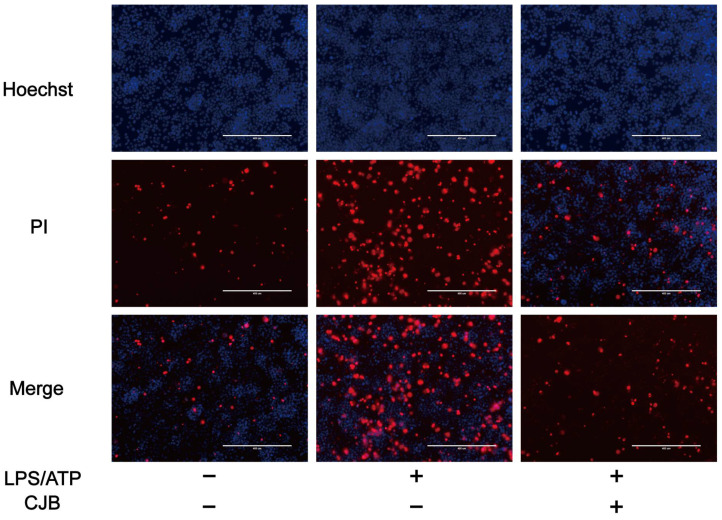
CJB maintained the cell membrane integrity. PI immunofluorescent staining to analyze cell membrane integrity: Hoechst (in blue), PI (in red), scale: 200 µm. “+” and “−” indicate whether the cell has been treated with the corresponding drug or not.

**Figure 3:**
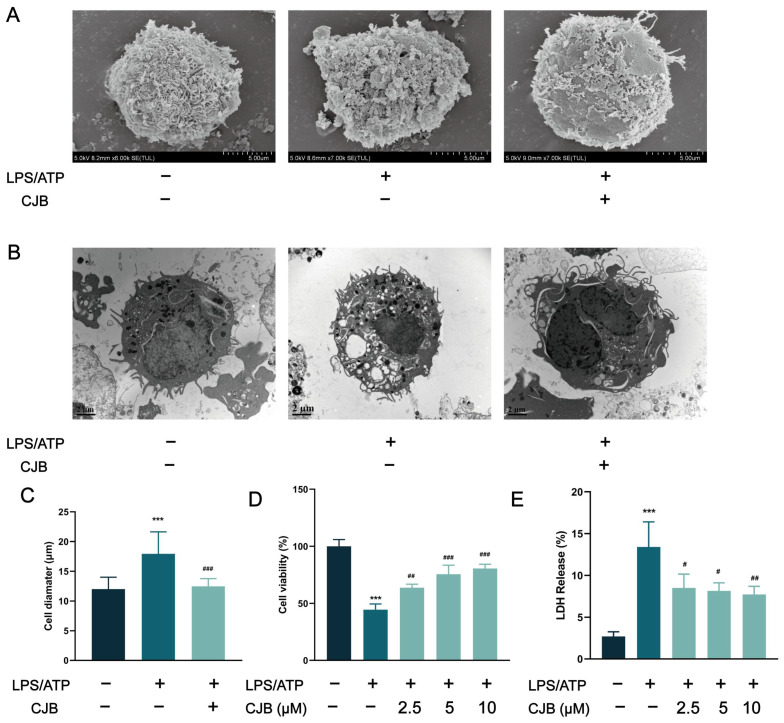
Anti-pyroptotic effect of CJB on THP-1 macrophage pyroptosis. (**A**) The cell morphology was analyzed using SEM. (**B**) The cell morphology was analyzed using TEM. (**C**) The cell diameter was measured using SEM, scale: 10 μm. (**D**) CCK8 assay for cell viability. (**E**) Detection of LDH content. The macrophages of control, LPS/ATP, and CJB treatment were colored dark gray, green, and light green, respectively. “+” and “−” indicate whether the cell has been treated with the corresponding drug or not. Data are expressed as mean ± SD (*n* = 3). *** *p* < 0.001 compared to the control group; ^#^ *p* < 0.05, ^##^ *p* < 0.01, and ^###^ *p* < 0.001 compared to the LPS/ATP group.

The structure of Chlojaponilactone B (CJB) is shown in Figure 1A. Initially, we employed the CCK8 assay to assess the impact of CJB on the viability of THP-1 macrophages. The results revealed that a concentration range of 1.25 µM to 20 µM of CJB had no toxic effects on THP-1 macrophages within a 24 h period (Figure 1B). Pyroptosis exhibited unique morphological characteristics. First, we utilized a propidium iodide (PI) staining assay to observe the integrity of the cell membrane. PI-positive cells increased remarkably after the stimulation of LPS/ATP, and CJB considerably reduced the PI-positive cells and maintained cell membrane integrity (Figure 2). Second, scanning electron microscopy (SEM) and transmission electron microscopy (TEM) were employed to detect morphological changes in THP-1 macrophages. Upon stimulation with LPS/ATP, THP-1 macrophages exhibited swelling and lysis, the formation of holes in the cell membrane, and the generation of vesicles within the cells. However, CJB could alleviate these morphological changes and maintain cell morphology (Figure 3A–C). Next, we found that cell viability decreased to 40% upon LPS/ATP stimulation, but with the increased concentration of CJB, cell viability was significantly increased (Figure 3D). Moreover, the LDH release increased markedly in the LPS/ATP group, while CJB attenuated this release (Figure 3E). According to the results, LPS/ATP induced cell swelling and lysis, LDH release, and cell viability decrease, indicating that the cells underwent pyroptosis [21,22]. Notably, CJB effectively inhibited the pyroptosis of THP-1 macrophages.

### 2.2. CJB Suppressed the NLRP3 Activation Stimulated by LPS/ATP

**Figure 4:**
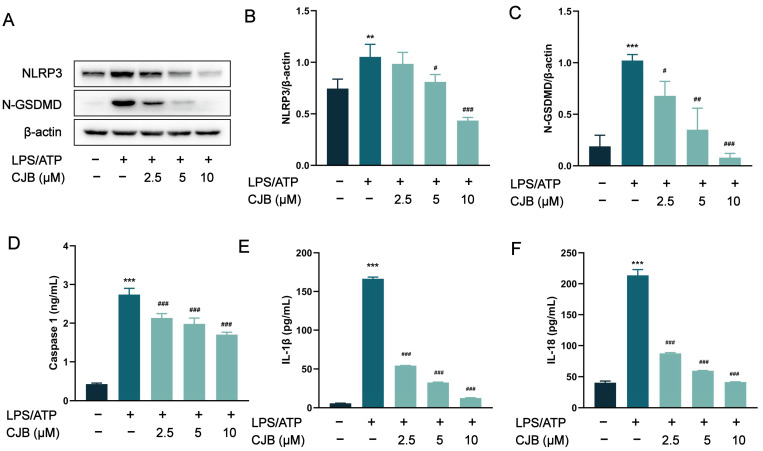
Effect of CJB on LPS/ATP-induced activation of NLRP3 in THP-1 macrophages. (**A**) The representative image of Western blot analysis of NLRP3 and N-GSDMD. (**B**,**C**) The quantification of expressions of NLRP3 and N-GSDMD relative to β-actin. (**D**) The production of caspase 1. (**E**,**F**) The cytokine release of IL-1β and IL-18. The macrophages of control, LPS/ATP, and CJB treatment are colored dark gray, green, and light green, respectively. “+” and “−” indicate whether the cell has been treated with the corresponding drug or not. Data are expressed as mean ± SD (*n* = 3). ** *p* < 0.01 and *** *p* < 0.001 compared to the control group; ^#^ *p* < 0.05, ^##^ *p* < 0.01, and ^###^ *p* < 0.001 compared to the LPS/ATP group.

To assess the impact of CJB on the activation of the canonical inflammasome NLRP3, we performed Western blot and ELISA experiments to detect the expression of its key components: NLRP3 and caspase 1. The protein levels of NLRP3, N-GSDMD, and caspase 1 were significantly increased, induced by LPS/ATP stimulation, but were concentration-dependently reduced by treatment with CJB (Figure 4A–D). As the executor of pyroptosis, GSDMD was cleaved by caspase 1 into N-GSDMD, and N-GSDMD perforated the cell membrane, leading to IL-18 and IL-1β moving through those holes, ultimately exacerbating the inflammatory response. CJB downregulated the protein expression of N-GSDMD, and significantly reduced the release of both IL-1β and IL-18 (Figure 4E,F). These results suggested that CJB inhibits the activation of NLRP3 in THP-1 macrophages, and suppresses the NLRP3/caspase 1 pyroptotic pathway.

### 2.3. CJB Interferes with the LPS/ATP-Induced Transcriptome Sequencing of THP-1 Macrophages

To better understand the effect of CJB treatment on pyroptosis and its molecular mechanism, the expression profiles of RNA alternations were constructed via an RNA-sequencing analysis of total RNA from the control macrophages (control), LPS/ATP-treated macrophages (model), and LPS/ATP plus CJB-treated macrophages (treatment). Differentially expressed genes (DEGs) were selected based on the criteria of q value < 0.05, and |fold change| > 2. Results showed that, compared to the control macrophages, LPS/ATP-treated macrophages exhibited 2342 DEGs, including 885 upregulated and 1457 downregulated genes. When compared to LPS/ATP-treated macrophages, the CJB-treated macrophages displayed 2351 DEGs, with 1273 upregulated and 1078 downregulated genes. 

**Figure 5:**
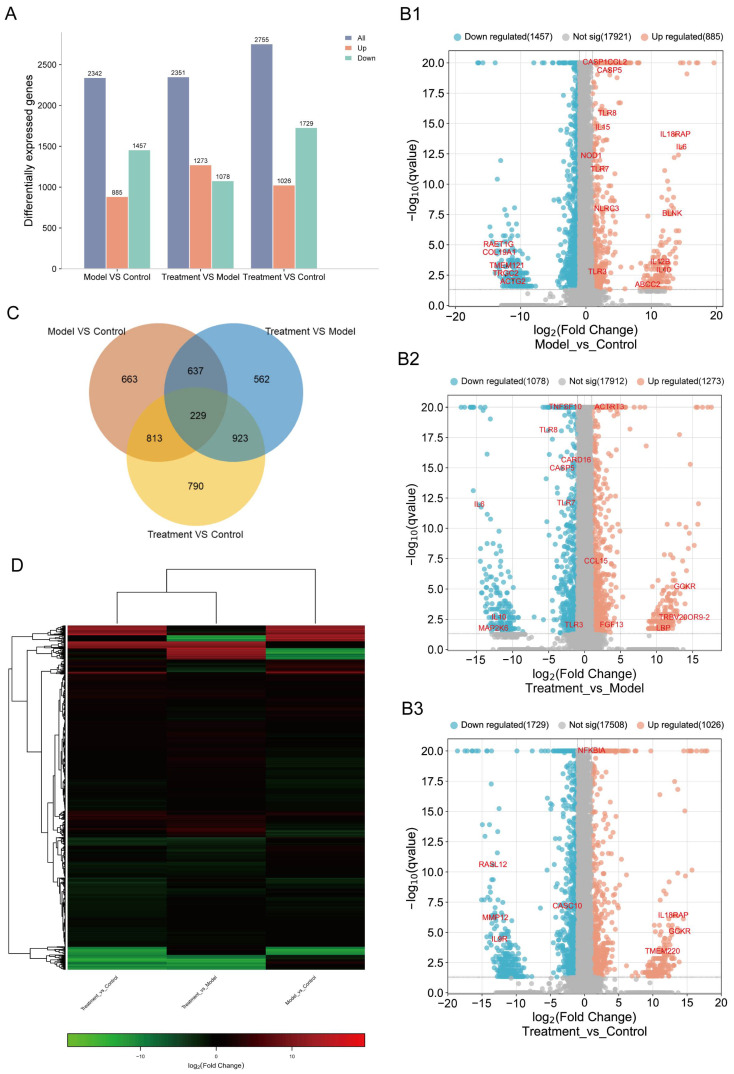
CJB treatment significantly altered the transcriptome profile of LPS/ATP-stimulated THP-1 macrophages. The transcriptome of control, LPS/ATP-stimulated macrophages, and CJB treated macrophages was constructed by RNA-seq. (**A**) Histogram of up- and downregulated genes. (**B1**–**B3**) Volcano plot of DEGs from control, model (stimulated with LPS/ATP), and treatment (LPS/ATP plus CJB treated) macrophages. (**C**) Venn diagram of DEGs. (**D**) Heatmap plotted of DEGs. The DEGs were selected based on one-way ANOVA (false discovery rate < 0.05), fold-change relative to control group (fc > 1.5). The color indicates the expression levels, from high (red) to low (green).

In contrast to the control, the CJB-treated macrophages showed 2755 DEGs, comprising 1026 up-regulated and 1729 down-regulated genes (Figure 5A). There were 663 of the same DEGs between the control and model, 562 of the same DEGs between the model and treatment, and 790 of the same DEGs between the control and treatment macrophages; 229 of the same DEGs were shared among the control, model, and treatment macrophages (Figure 5C). The volcano plots visually represent these results. Among the shared DEGs, several DEGs were screened based on the comparison between the treatment and the model, including CASP5, NOD1, IL18RAP, IL6, IL12B, TLR3, TLR7, and TLR8 (Figure 5B1–B3). These genes belong to caspases, nod-like receptors, interleukins, and Toll-like receptors. These results demonstrated that CJB reduced the transcription and translation of these key genes, thereby influencing the process of THP-1 macrophage pyroptosis and inflammation. Figure 5D depicts the heatmap showing the distribution of DEGs within each group. All the DEGs were utilized in the subsequent analyses.

### 2.4. DEG Profiles and Functional Prediction Highlighted That CJB Suppressed LPS/ATP-Induced THP-1 Macrophages’ Pyroptosis through the TLR/NF-κB Signaling Pathway

**Figure 6:**
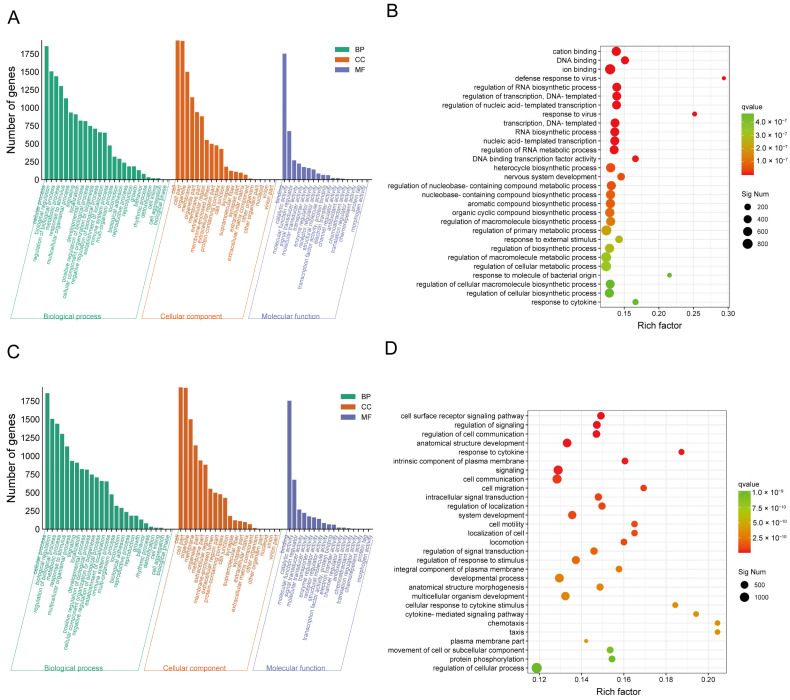
GO analysis of DEGs highlights that CJB suppresses LPS/ATP-induced THP-1 macrophages’ pyroptosis through the TLR/NF-κB signaling pathway. (**A**,**B**): GO analysis of the DEGs between the model and control. (**C**,**D**): GO analysis of the DEGs between the treatment and model. GO annotation of the DEGs and GO bubble diagram of DEGs, including the categories of molecular functions (MF), cellular components (CC), and biological processes (BP). Qvalue is indicated by color, and the number of differential genes contained under each function is indicated by the size of the dot. The top 30 most-enriched GOs are highlighted.

Gene ontology (GO) and Kyoto Encyclopedia of Genes and Genomes (KEGG) annotation-based enrichment analyses were conducted to illustrate the function of the aforementioned DEGs. The results of GO enrichment analysis revealed that the DEGs were primarily enriched in the cellular components category. When comparing the LPS/ATP-treated macrophages to the control macrophages, the DEGs were significantly engaged in response to molecules of bacterial origin, the defense response to viruses, the response to cytokine, and the regulation of primary metabolic process (Figure 6A,B). Similarly, when comparing the CJB-treated macrophages to the LPS/ATP-treated macrophages, the DEGs were significantly involved in the cell surface receptor signaling pathway, signaling, the response to cytokines, and cell communication (Figure 6C,D).

**Figure 7:**
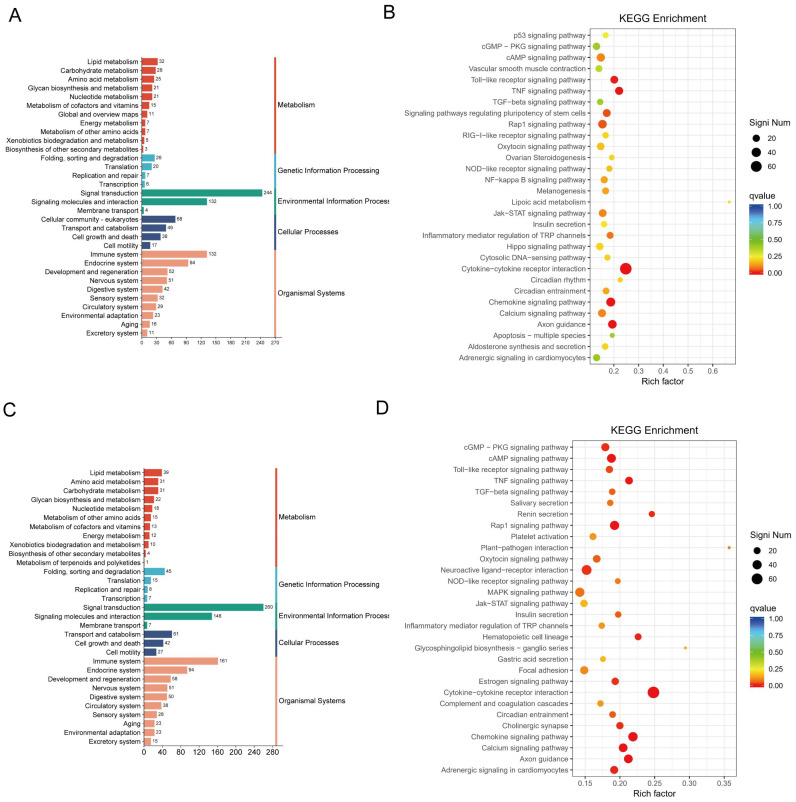
Enrichment analysis of DEGs with KEGG annotations. (**A**,**B**): KEGG pathway classification map and enrichment analysis of DEGs between model and control. (**C**,**D**): KEGG pathway classification map and enrichment analysis of DEGs between treatment and model. KEGG pathway classification map and KEGG bubble diagram of DEGs. Qvalue is indicated by color, and the number of differential genes contained under each function is indicated by the size of the dot. The top 30 most enriched signaling pathways were highlighted.

To further explore the interactions among DEGs following CJB treatment, we annotated and enriched the DEGs using the KEGG biological pathways database. As for KEGG analysis, the DEGs in the model compared to the control were highly enriched in cytokine–cytokine receptor interaction, the TNF signaling pathway, the inflammatory mediator regulation of transient receptor potential (TRP) channels, and the Toll-like receptor signaling pathway (Figure 7A,B). Interestingly, we discovered that the DEGs between the treatment and the model were also significantly enriched in cytokine–cytokine receptor interaction, the chemokine signaling pathway, the inflammatory mediator regulation of TRP channels, and the Toll-like receptor signaling pathway (Figure 7C,D). These observations indicate that the CJB may exert anti-inflammatory and anti-pyroptotic effects, primarily by suppressing the cytokine–cytokine receptor interaction, TNF signaling pathway, Toll-like receptor signaling pathway, and NF-κB signaling pathway.

### 2.5. CJB Suppressed the TLR/NF-κB Signaling Pathway

**Figure 8:**
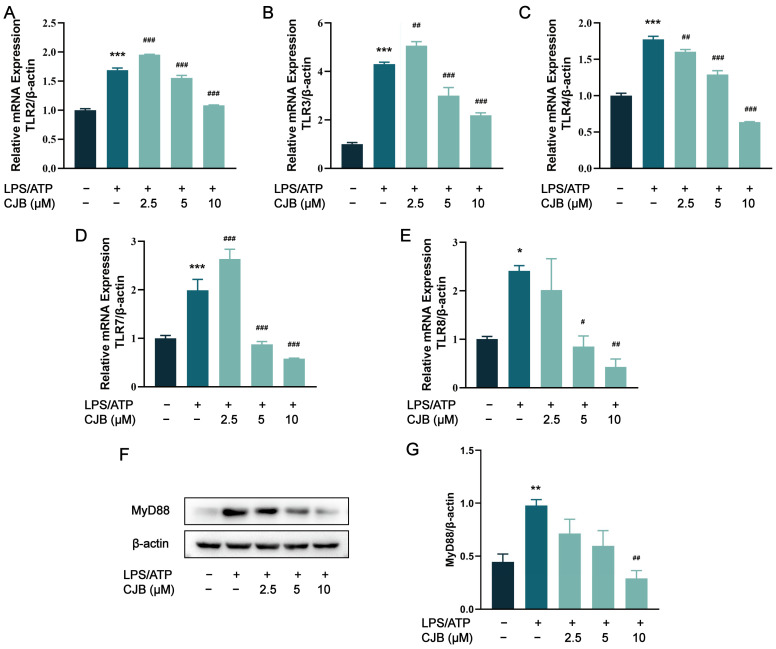
CJB decreased the expression of TLRs and MyD88. (**A**–**E**) The mRNA expression of TLR2, TLR3, TLR4, TLR7, and TLR8. (**F**) The representative image of the Western blot analysis of MyD88. (**G**) The quantification of expressions of MyD88 relative to β-actin. The macrophages of control, LPS/ATP, and CJB treatment are colored dark gray, green, and light green, respectively. “+” and “−” indicate whether the cell was treated with the corresponding drug or not. Data are expressed as mean ± SD (*n* = 3). * *p* < 0.05, ** *p* < 0.01, and *** *p* < 0.001 compared to the control group; ^#^ *p* < 0.05, ^##^ *p* < 0.01, and ^###^ *p* < 0.001 compared to the LPS/ATP group.

To further investigate whether CJB influenced the TLR-mediated activation of the NF-κB signaling pathway, we analyzed the mRNA or protein levels of TLRs, MyD88, and NF-κB (LPS/ATP was used as a positive control). As depicted in Figure 8A–E, the stimulation of LPS/ATP led to upregulated mRNA levels for TLR2, 3, 4, 7, and 8, while treatment with 5 and 10 µM CJB exerted an inhibitory effect. MyD88 serves as a junction protein in the TLR/MyD88/NF-κB signaling pathway, mediating the downstream signal transduction during LPS stimulation. The results obtained from Western blot analysis demonstrated that CJB reduced the elevated expression of MyD88 induced by LPS/ATP in a concentration-dependent manner (Figure 8F,G). 

**Figure 9:**
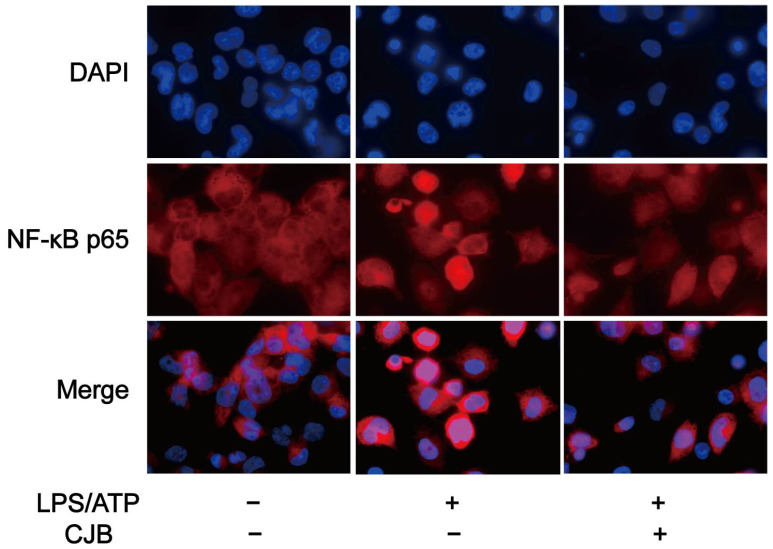
CJB attenuated the NF-κB nuclear translocation of p65 caused by LPS/ATP treatment. Immunofluorescence staining of NF-κB p65 nuclear translocation; p65 (in red), DAPI (in blue). “+” and “−” indicate whether the cell was treated with the corresponding drug or not.

**Figure 10:**
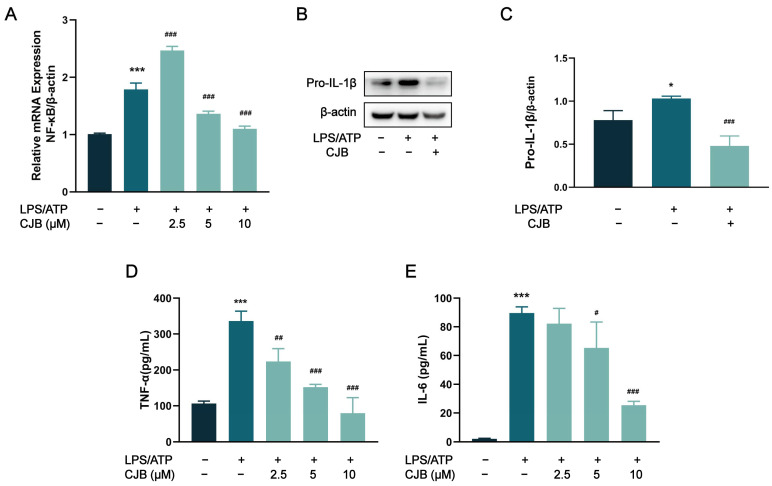
CJB decreased the production of pro-IL-1β, TNF-α, and IL-6 via the inhibition of the NF-κB pathway. (**A**) The mRNA level of NF-κB. (**B**) The representative image of the Western blot analysis of pro-IL-β. (**C**) The quantification of expressions of pro-IL-β relative to β-actin. (**D**,**E**) ELISA was performed to detect the release of TNF-α and IL-6. The macrophages of control, LPS/ATP, and CJB treatment are colored dark gray, green, and light green, respectively. “+” and “−” indicate whether the cell was treated with the corresponding drug or not. Data are presented as mean ± SD (*n* = 3). * *p* < 0.05 and *** *p* < 0.001 compared to the control group; ^#^ *p* < 0.05, ^##^ *p* < 0.01, and ^###^ *p* < 0.001 compared with the LPS/ATP group.

Previous studies have shown that NF-κB is involved in the mechanism of pyroptosis and regulates the expression of multiple chemokines, including IL-1β, IL-6, and TNF-α [23]. To investigate the potential effect of CJB on the NF-κB signaling pathway, qRT-PCR and immuno-fluorescence staining were performed to explore the expression and activation of NF-κB. The results demonstrated that CJB attenuated the nuclear translocation of p65 induced by LPS/ATP treatment (Figure 9) and downregulated the mRNA level of NF-κB (Figure 10A). Furthermore, we evaluated the effects of CJB on the production of pro-IL-1β, TNF-α, and IL-6. CJB suppressed the protein expressions of pro-IL-1β (Figure 10B). Additionally, ELISA results revealed that the production of TNF-α and IL-6 was increased following LPS/ATP stimulation, while treatment with CJB elicited a concentration-dependent decrease (Figure 10D,E). 

## 3. Discussion

Extensive research has demonstrated that pyroptosis, a crucial pathological process, plays a significant role in the development of numerous inflammatory diseases, including myocarditis, atherosclerosis, and arthritis. The activation of inflammation and pyroptosis exacerbates the progression of viral myocarditis [24]. Similarly, exposure to polybrominated diphenyl ethers promotes atherosclerosis through lipid accumulation, NLRP3 inflammasome activation, and pyroptosis [12]. These findings suggest that inhibiting pyroptosis may be an effective strategy to alleviate inflammatory conditions. For instance, targeting caspase 11–GSDMD-mediated pyroptosis is considered a potential therapeutic approach for atherosclerosis [25]. Additionally, cholecalciferol cholesterol emulsion has been found to possess anti-pyroptotic effects, reducing the severity of autoimmune myocarditis in mice [26]. In rheumatoid arthritis, several bioactive molecules, such as punicalagin, complement C1q, and quercetin, have been shown to regulate pyroptosis, thereby influencing disease progression [27,28,29,30]. 

*Chloranthus japonicus*, a traditional Chinese medicine, is widely used for treating colds, coughs, rheumatism, bruises, swelling, and pain [31]. CJB, a lindenane-type sesquiterpenoid lactone isolated from *Chloranthus japonicus*, has demonstrated good anti-inflammatory and antioxidant properties in previous studies. These properties are partially attributed to its inhibitory effect on NF-κB signaling pathway activation and its suppression of LPS-induced NO and inflammatory cytokine release (such as TNF-α, IL-6, iNOS) from RAW264.7 macrophages [19,20]. To further investigate the structure–activity relationship of CJB, we subjected it to perhydrogenation, resulting in a new compound, Perhydrochlojaponilactone B, which exhibits protective effects against hydrogen peroxide-induced oxidative damage in PC12 cells [32]. Given the significant role of pyroptosis in inflammatory responses, we hypothesized that CJB may play an important role in regulating pyroptosis. Our research findings demonstrate that CJB exhibits good anti-pyroptotic activity at a concentration of 10 μM, outperforming other known anti-pyroptotic molecules such as luteolin and quercetin [33,34]. These discoveries not only support the anti-inflammatory effects of CJB, but also emphasize its significance in regulating pyroptosis. The current study offers a novel theoretical foundation for the utilization of CJB in treating inflammatory disorders, establishing it as a promising contender for the development of anti-pyroptotic drugs. 

The core process of cell pyroptosis involves the activation of inflammatory caspases, particularly the NLRP3 inflammasome [35]. In this study, we delved into the effects of CJB on NLRP3 inflammasome activation and cell pyroptosis, revealing its underlying molecular mechanisms. NLRP3, a crucial component of innate immunity, forms an inflammasome in response to pathogens or danger signals, further activating caspase-1 [36]. This leads to the cleavage of GSDMD and its translocation to the cell membrane, forming pores and triggering pyroptosis. Our experimental data clearly demonstrated that CJB inhibited the activation of NLRP3 concentration-dependently, and also reduced the protein expression of N-GSDMD, caspase 1. This finding proved that CJB inhibits macrophages’ pyroptosis through the canonical pathway. Notably, the NLRP3 inflammasome plays a pivotal role in various diseases, including Alzheimer’s disease, type 2 diabetes, atherosclerosis, and cancer [37,38,39,40,41]. Therefore, CJB’s inhibitory effect on NLRP3 may provide new therapeutic avenues for these diseases. Furthermore, our study also found that CJB can reduce the secretion of IL-1β and IL-18, further validating its efficacy in suppressing inflammatory responses. 

For a further exploration of the effect of CJB treatment on pyroptosis and its molecular mechanism, RNA-seq analysis was performed on THP-1 macrophages treated with LPS/ATP only or with the addition of 10 µM CJB. Through GO and KEGG enrichment analysis, we observed significant changes in the expression levels of inflammation-related genes and receptor genes in THP-1 macrophages upon CJB treatment. The dates informed notable variations in the expression levels of genes that belonged to inflammatory pathways and receptors, such as Toll-like receptor, NOD-like receptor, nuclear factor kappa-B (NF-κB), the inflammatory mediator regulation of TRP channels, and tumor necrosis factor. The TRP channels are a cation-permeable channel superfamily expressed in mammalian cells and which participate in various pathological and physiological processes including pain, temperature, and mechanical sensation [42]. Multiple members of the TRP subfamily play crucial roles in immune and inflammatory diseases [43]. In particular, TRPM2, a member of the TRPM family, can respond to oxidative stress, TNF-α, and β-amyloid peptides [44]. Previous studies have shown that TNF-α can enhance the expression and activity of TRPA1, TRPV1, and TRPV4 [45,46]. Therefore, we speculate that the activation of the TNF signaling pathway during pyroptosis may lead to an increase in TRP channel function, thereby exacerbating inflammation and pyroptosis. CJB’s therapeutic effect may be achieved by interfering with this pathway and inhibiting TRP channel function, thereby mitigating inflammation and pyroptosis. 

Toll-like receptors act as sentinels, monitoring and recognizing various pathogen-associated molecular patterns and damage-associated molecular patterns, such as bacteria, viruses, and endotoxins [47]. They activate innate immunity and acquired immunity, thereby promoting the elimination of pathogens and regulating disease progression, and also play an important role in pyroptosis and inflammation [48,49]. For one thing, TLR stimulation often acts in cooperation with TLRs and caspase 1-activating NLRs, leading to an increased vulnerability to NLR-mediated caspase 1 activation after ATP treatment [35]. For another, the TLR/NF-κB pathway mediates the increase in NLRP3 and pro-IL-1β. Among the DEGs, CJB reduced the gene expression of Toll-like receptors (TLRs) TLR3, TLR7, and TLR8. TLR3 generally plays a role in antiviral and innate immune responses [50]. TLR7 is widely distributed in immune cells, which can bind to MyD88 to initiate downstream inflammatory cascade responses, and is involved in rheumatoid arthritis progression [51,52,53]. TLR8 is a vital member of the TLR family, exerting antiviral and anti-bacterial effects [54,55]. Activated TLR8 initiates a MyD88-dependent signaling pathway that mediates the immune response, and results in the secretion of pro-inflammatory cytokines such as TNF-α, IFN-γ, and IL-12 [56,57]. Our RNA-sequencing results also showed that CJB reduced LPS/ATP-induced IL-12B gene expression. Much research has indicated the role of TLR2 and TLR4 in inflammatory diseases; their most notable biological function is to directly or indirectly promote the production and release of inflammatory factors, exerting major antimicrobial and anti-inflammatory effects [58,59]. Both of them could recognize LPS and then trigger the inflammatory response [60,61]. Therefore, to verify that the CJB affects the TLR pathway, qRT-PCR was used to evaluate the expression of the TLR2,3,4,7,8 in macrophages. The results showed that 5 or 10 µM CJB could decrease LPS/ATP-induced elevated levels of the above Toll-like receptor expression in macrophages. The TLR signaling pathways include MyD88-dependent and MyD88 non-dependent pathways, and the PI3K signaling pathway [62,63,64]. MyD88 is a pivotal connector protein in the TLR signaling pathway. TLRs trigger MyD88-dependent and non-dependent pathways by recognizing different ligands, leading to the activation of pro-inflammatory cytokines and co-stimulatory molecules, thus inducing inflammatory responses [65]. We further examined the expression of MyD88; the result showed that CJB reduced its protein expression. Our results indicate that CJB inhibited THP-1 macrophages’ pyroptosis by inhibiting the TLR/MyD88 signaling pathway.

Previous research has proved that NLRP3 activation is a two-step process. In the first step, Toll-like receptors (TLRs) bind to ligands such as LPS, recruiting downstream signaling receptor proteins such as adapter proteins containing TIR domains and MyD88, or through cytokines such as TNF and IL-1β that lead to NF-κB activation and NLRP3 pro-IL-1β gene transcription. In the second step, NLRP3 is activated by various stimuli, including ATP, RNA viruses, pore-forming toxins, etc. [23,66] 

Stimulation by TLRs or cytokines triggers the activation of NF-κB and gene transcription, subsequently upregulating the expression of NLRP3. TLR stimulation with extracellular ATP can rapidly activate NLRP3 [67]. There is an interaction between the NF-κB signaling pathway and inflammatory cytokines. Activated NF-κB can promote the transcription of inflammatory factor genes. Inflammatory factors can also enhance their expression by activating the NF-κB signaling pathway [68,69]. Therefore, the activation of NLRP3 involves complex interactions among TLRs, cytokines, and the NF-κB signaling pathway, leading to the upregulation of inflammatory genes such as NLRP3 and IL-1β. Most findings suggest that NF-κB upregulation can activate the NF-κB signaling pathway and the NLRP3 inflammasome [70,71,72]. Both the NF-κB signaling pathway and the NLRP3 inflammasome can exacerbate pyroptosis. Therefore, we measured NF-κB nuclear translocation along with NF-κB mRNA expression. Our results demonstrated that CJB decreases the mRNA expression of NF-κB and attenuates the nuclear translocation of p65. Generally, the inactivated NF-κB protein is a trimeric complex composed of p65, p50, and IκB and distributed in the cytoplasm [73]. When the upstream signal molecule has been delivered to the IκB kinase, the IκB will phosphorylate and dissociate, enabling the NF-κB dimer to be transferred to the nucleus. Activation of the NF-κB pathway promotes the production of pro-IL-1β and the secretion of pro-inflammatory cytokines TNF-α, IL-6, and IL-1β [74,75]. Our data showed that the CJB-attenuated nuclear translocation of p65 further decreased pro-IL-1β production, and decreased the secretion of TNF-α and IL-6. These findings indicate that CJB prevents pyroptosis by inactivating NF-κB. Additionally, our results suggest that CJB attenuated the pyroptosis of THP-1 macrophages by downregulating the TLR/NF-κB pathway. Notably, at a low concentration of 2.5 µM, CJB caused the increased expression of TLR2, 3, 7, and NF-κB. However, TLR3 and TLR7 are not involved in LPS/ATP-induced pyroptosis. Therefore, we speculate that these nonspecific increases in mRNA expression may be attributed to the cellular stress response. The underlying reasons for this observation require further investigation.

Overall, our findings provide valuable insights into the molecular mechanisms underlying the inhibitory effects of CJB on NLRP3 inflammasome activation and pyroptosis. Future studies are warranted to further elucidate the precise role of CJB in regulating inflammatory responses and its therapeutic potential in various diseases.

## 4. Materials and Methods

### 4.1. Reagents and Antibodies

The Adenosine 5′-triphosphate disodium salt (ATP), lipopolysaccharide (LPS), and phorbol myristate acetate (PMA) were purchased from Sigma-Aldrich (Shanghai, China). Antibodies against NLRP3 (D4D8T), GSDMD (E5O4N), β-actin (8H10D10), and MyD88 (D80F5), and anti-rabbit IgG (7074S) and anti-mouse IgG (7076S) HRP-linked antibodies, were obtained from Cell Signaling Technology (Boston, MA, USA). The antibody against IL-1β (EPR21086) was acquired from Abcam (Cambridge, UK). Mouse ELISA kits (IL-1β, IL-18, TNF-α, and IL-6) and a human ELISA kit (caspase 1) were purchased from Neobioscience (Shenzhen, China).

### 4.2. Material

The air-dried plants of *Chloranthus japonicus* were powdered and extracted using 95% EtOH at room temperature to obtain a crude extract. The crude extract was suspended in H_2_O and fractionated sequentially with petroleum ether, EtOAc, and n-BuOH, and CJB was isolated from the EtOAc extract [20]. The detailed isolation and purification methods and NMR (^1^H, ^13^C) spectra of CJB were recorded in the Supplementary Material (Figures S1–S3). The material under study is endotoxin-free.

### 4.3. Cell Culture and Induction

THP-1 cells were from The Cell Bank of Shanghai Institute of Biochemistry and Cell Biology (Shanghai, China). The cells were cultured in RPMI medium 1640 (Gibco, NY, USA) containing 10% fetal bovine serum (Gibco, NY, USA) and 1% penicillin/streptomycin (Gibco, NY, USA), in a 5% CO_2_, 37 °C incubator. The THP-1 macrophage pyroptosis model was induced according to the previously published procedure [33,34,76]. THP-1 cells were plated in 6-well culture plates (1 × 10^6^ cells/well), and differentiated into macrophages after 24 h of 100 nM PMA stimulation. The macrophages were treated with LPS (100 ng/mL) only or LPS (100 ng/mL) plus CJB (2.5, 5, and 10 μM) for 5 h, and then exposed to ATP (5 mM) for 1 h. 

### 4.4. Cell Viability Assay

The cells were seeded into the wells of 96-well plates (1 × 10^4^ cells/mL) and cultured overnight. After 24 h treatment at different concentrations (40, 20, 10, 5, and 2.5 μM) of CJB, 10 μL CCK8 solution (Beyotime, Shanghai, China) was added to the wells and incubated at 37 °C for 2 h. The absorbance at 450 nm was detected by a microplate reader (Synergy H1, BioTek, Winooski, VT, USA).

### 4.5. LDH Assay

An LDH assay kit (Beyotime, Shanghai, China) was utilized to measure the LDH produced by THP-1 macrophages. After LPS/ATP with or without CJB treatment, 20 μL of LDH release reagent was added to the maximum LDH release control wells and mixed well, and then left to continue incubating for 1 h. After that, 120 μL cell supernatants were mixed with 60 μL working solution, incubated for 30 min at room temperature away from light, and the absorbance subsequently measured at 450 nm.

### 4.6. Propidium Iodide Staining

Hoechst and PI dye solutions (Beyotime, Shanghai, China) were used to stain THP-1 macrophages. After a PBS wash, the cells were stained for 30 min at 4 °C using 5 µL PI stain and 5 µL Hoechst 33342 stain. Then, the cells were washed with PBS and viewed under a fluorescence microscope (EVOS FL Auto, Thermo, Waltham, MA, USA).

### 4.7. Western Blotting 

RIPA mixed with 1% PMSF (Beyotime, Shanghai, China) was used to extract total proteins of THP-1 macrophages. The concentration of proteins was measured using a BCA kit (Beyotime, Shanghai, China). After being quantified to 40 μg, proteins were separated by SDS-PAGE and transmitted into PVDF membranes (Millipore, MA, USA). Then, 5% skim milk (Biofroxx, Thuringia, Germany) was used to block membranes for 1 h, and then they were incubated with primary antibody at 4 °C overnight, and then incubated with secondary antibodies for 1.5 h at room temperature. Protein bands were detected with Western Chemiluminescent HRP Substrate (Millipore, MA, USA), and the image was captured using the 5200CE Tanon™ chemi-image system (Tanon, Shanghai, China). 

### 4.8. ELISA Assay 

THP-1 macrophages were seeded in a 6-well plate (1.2 × 10^6^ cells/well). After treatment with LPS (100 ng/mL) only or LPS (100 ng/mL) plus CJB (2.5, 5, and 10 μM) for 5 h, and then exposure to ATP (5 mM) for 1 h, the cell supernatant was collected to detect cytokine levels. The experiment was performed using commercial ELISA kits in accordance with the instructions of the manufacturer.

### 4.9. NF-κB p65 Nuclear Translocation

The cells were seeded in confocal dishes. After treatment, the cells were fixed in 4% paraformaldehyde for 20 min, and 0.5% TritonX-100 was used to punch cell membranes. It was blocked with 5% BSA for 30 min at room temperature, and then incubated with NF-κB p65 antibody (diluted 1:100, Beyotime, China) at 4 °C overnight, followed by Cy3-labeled anti-mouse IgG (diluted 1:500, Beyotime, China) for 1 h at room temperature. Ultimately, the nuclei were stained with DAPI (1 μg/mL) for 5 min and observed using a laser scanning confocal microscope (FV3000, Olympus, Tokyo, Japan). 

### 4.10. RNA-Seq Analysis

The total RNA of macrophages was extracted with TRIeasyTM total RNA extraction reagent (Yeasen, Shanghai, China), and RNA sequencing was carried out on an Illumina platform from Sangon Biotech (Guangzhou, China). Differentially expressed genes (DEGs) were identified according to the criteria of fold change >1.5 and *p* values < 0.05, followed by GO and KEGG analyses. 

### 4.11. qRT-PCR

A Nanodrop 2000 ultramicro spectrophotometer (Thermo Fisher Scientific, Waltham, MA, USA) was used to detect the RNA concentration. The reverse transcription of RNA to obtain cDNA by using 1st Strand cDNA Synthesis SuperMix for qPCR (Yeasen, Shanghai, China) and qRT-PCR was performed by using a qPCR SYBR Green Master Mix (Yeasen, Shanghai, China) in a LightCycler 480Ⅱ PCR System (Roche, Basel, Switzerland). The reaction procedure was as follows: preincubated at 95 °C for 5 min, 40 cycles of deformation at 95 °C for 10 s, annealing at different temperatures (55 or 60 °C) for 20 s, and extension at 72 °C for 20 s. β-actin served as a reference gene, and the experiment was carried out in three biological replicates. Oligonucleotide primers are listed in Table 1.

### 4.12. Statistical Analysis

Results were presented as mean ± SD (*n* = 3), and the data were analyzed using GraphPad Prism 8.0 software (GraphPad Software, Boston, MA, USA). One-way ANOVA was used for a variety of comparative statistical analysis, followed by Dunnett’s test. Values of *p* < 0.05 are considered significant.

## 5. Conclusions

CJB effectively attenuated THP-1 macrophage pyroptosis triggered by LPS/ATP. We established that CJB inhibited NLRP3 inflammasome activation and anti-pyroptotic effects by suppressing the TLR/MyD88/NF-κB pathway. These findings suggest that CJB has an outstanding anti-pyroptotic effect and plays an anti-inflammatory role through anti-pyroptosis, thus providing a potential bioactive chemical for the prevention and treatment of pyroptosis-related inflammatory diseases. Furthermore, our study provides a scientific basis for the development of anti-inflammatory drugs derived from the natural products of the lindenane-type sesquiterpenoid lactone.

## Figures and Tables

**Table 1:** Primer sequences of the targeted genes.

Gene Symbol	Primer Sequences
β-actin	F 5′-CACGAAACTACCTTCAACTCC-3′ R 5′-CATACTCCTGCTTGCTGATC-3′
TLR2	F 5′-TCCGTCTTTTTGATGAGAACAATG-3′ F 5′-ACTCCAGGTAGGTCTTGGTGTTCA-3′
TLR3	F 5′-AGTGCCGTCTATTTGCCACA-3′ F 5′-GCATCCCAAAGGGCAAAAGG-3′
TLR4	F 5′-TGCTTCTTGCTGGCTGCATA-3′ F 5′-CCAGTCCTCATCCTGGCTTG-3′
TLR7	F 5′-CACAGCCGTCCCTACTGTTT-3′ F 5′-TTTTTACACGGCGCACAAGG-3′
TLR8	F 5′-ACATCAGCAAGACCCATCCC-3′ F 5′-TCTTCGGCGCATAACTCACA-3′
NF-κB	F 5′-TGCAGCAGACCAAGGAGATG-3′ F 5′-TGCATTGGGGGCTTTACTGT-3′

## Data Availability

Data are contained within the article and Supplementary Materials.

## Supplementary Materials

The following supporting information can be downloaded at: https://www.mdpi.com/article/10.3390/ph17030402/s1, Figure S1: Isolation and purification of Chlojaponilactone B; Figure S2: ^1^H NMR spectrum of Chlojaponilactone B (CDC_l3_, 400 MHz); Figure S3: ^13^C NMR spectrum of Chlojaponilactone B (CDC_l3_, 100 MHz).

## Abbreviations

CJB, Chlojaponilactone B; LPS, lipopolysaccharides; PMA, phorbol myristate acetate; PI, LDH, lactate dehydrogenase; NLRP3, NOD-like receptor protein 3; GSDMD, Gasdermin D; ASC, apoptosis-related speck-like protein; TLRs, Toll-like receptors; MyD88, myeloid differentiation factor 88; NF-κB, nuclear factor κB; TNF-α, tumor necrosis factor-alpha; MAPK, mitogen-activated protein kinase; PI3K, phosphatidylinositol 3-kinase; IFN-γ, Interferon-γ; IL-6, Interleukin-6; IL-18, Interleukin-18;IL-1β, Interleukin-1β; SEM, scanning electron microscope; TEM, transmission electron microscope; DEGs, differentially expressed genes; KEGG, Kyoto Encyclopedia of Genes and Genomes; GO, gene ontology; TRP channel, transient receptor potential channel.
