# Supplementary material for: Chlojaponilactone B Attenuates THP-1 Macrophage Pyroptosis by Inhibiting the TLR/MyD88/NF-κB Pathway
Source: Pharmaceuticals (Basel). 2024 Mar 21;17(3):402. doi: 10.3390/ph17030402 (PMC10975068; doi:10.3390/ph17030402)
Supplement: Supplementary file 1 [file pharmaceuticals-17-00402-s001.zip › pharmaceuticals-2889539-supplementary.pdf]

Chojaponilactone B attenuates THP-1 macrophage pyroptosis by inhibiting TLR/MyD88/NF-κB pathway

Qinyin Wen [1], Bingjinfeng Zhan [1], Lu Jin [1], Zijing Peng [1], Ju Liu [1], Longping Zhu [1], Depo Yang [1], Xinjun Xu [1], Lixia Zhang [2], Ge Li [2] and Zhimin, Zhao[1,*]

[1] School of Pharmaceutical Sciences, Sun Yat-sen University, Guangzhou, Guangdong, China.

[2] Yunnan Key Laboratory of Southern Medicine Utilization,Yunnan Branch Institute of Medicinal Plant Development, Chinese Academy of Medical Sciences, Jinghong, China.

*Correspondence: Zhimin Zhao: zhaozhm2@mail.sysu.edu.cn; Tel.: +86-020-39943043

Figure	content	page
S1	Isolation and purification of Chlojaponilactone B	3
S2	^{1}H NMR spectrum of Chlojaponilactone B	4
S3	^{13}C NMR spectrum of Chlojaponilactone B	5

S1. Isolation and purification of Chlojaponilactone B

The whole plants of *Chloranthus japonicus* were collected in March 2018 at Xishuangbanna, Yunnan Province, China, and authenticated by one of the co-authors (Depo, Yang). A voucher specimen (accession number: CJB201806) has been deposited at the School of Pharmaceutical Sciences, Sun Yat-sen University. Whole *Chloranthus japonicus* plants (20 kg) were air-dried, powdered, and extracted using 95% EtOH (3 × 75 L) for 1 week each at room temperature. The 95% EtOH extract was evaporatedby removing the solvents to obtain the crude extract (1.5 kg), which was suspended in H2O (5 L). The crude extract was partitioned into petroleum ether (3 × 5 L), EtOAc (3 × 5 L), and *n*-BuOH (3 × 5 L) fractions. The EtOAc extract (485.00 g) was purified by MCI gel column chromatography (CC) by gradient elution with an MeOH/H2O gradient (30:70 to 100:0) to obtain five fractions (I-V). Fraction III (52.06 g) was chromatographed over a C18 reversed-phase (RP-18) silica gel by gradient elution with petroleum ether/EtOAc (100:0 to 75:25) to obtain six fractions (IIIa-IIIf). Fraction IIIc (1.95 g) was purified on a Sephadex LH-20 CC column using isocratic elution with CH2Cl2/MeOH (0:100) to afford three fractions (IVa–IVc). Finally, Fraction IVb (700 mg) was purified by HPLC with MeOH/H2O (70:30) to yield Chlojaponilactone B (300 mg).

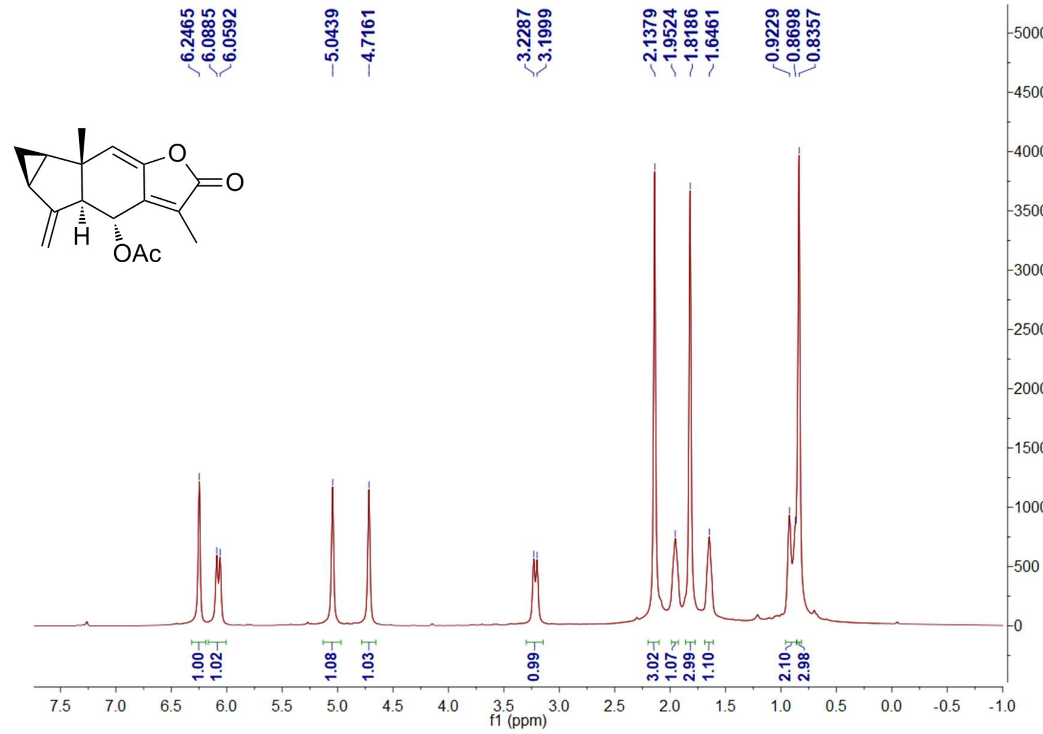

S2. ^{1}H NMR spectrum of Chlojaponilactone B (CDCl$_3$, 400 MHz)

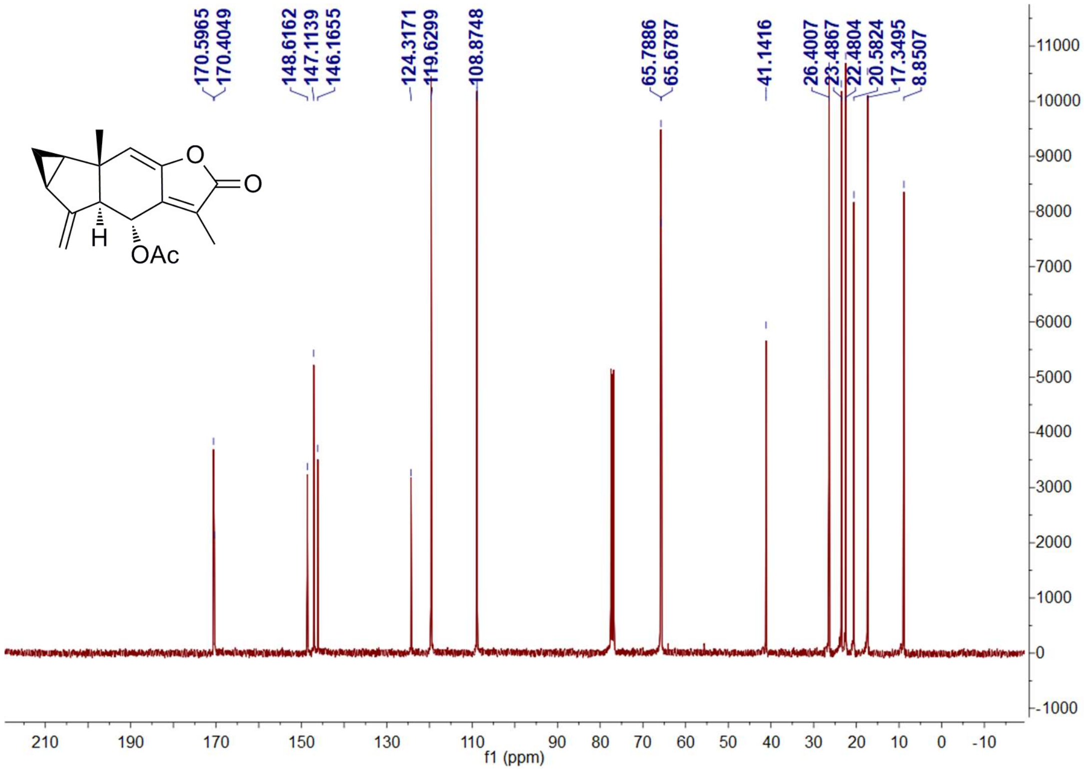

S3. ^{13}C NMR spectrum of Chlojaponilactone B ($CDCl_3$, 100 MHz)
